# Preparation and Characterization of Hydrophilically Modified PVDF Membranes by a Novel Nonsolvent Thermally Induced Phase Separation Method

**DOI:** 10.3390/membranes6040047

**Published:** 2016-11-18

**Authors:** Ningen Hu, Tonghu Xiao, Xinhai Cai, Lining Ding, Yuhua Fu, Xing Yang

**Affiliations:** 1 Faculty of Materials Science and Chemical Engineering, Ningbo University, Ningbo 315211, China; huningen@163.com (N.H.); caixinhai@ytu.edu.cn (X.C.); fuyuhuafyh@163.com (Y.F.); 2 Tri-Tech Chemical Co. Pty Ltd, 5-11 Normanby Ave, Sunshine VIC 3020, Australia; lining@ttcc.com.au; 3 Institute for Sustainability and Innovation, College of Engineering and Science, Victoria University, P.O. Box 14428, Melbourne 8001, Australia

**Keywords:** nonsolvent thermally-induced phase separation (NTIPS), PVDF/PVA blend membrane, hydrophilic modification, membrane resistance, antifouling property

## Abstract

In this study, a nonsolvent thermally-induced phase separation (NTIPS) method was first proposed to fabricate hydrophilically-modified poly(vinylidene fluoride) (PVDF) membranes to overcome the drawbacks of conventional thermally-induced phase separation (TIPS) and nonsolvent-induced phase separation (NIPS) methods. Hydrophilically-modified PVDF membranes were successfully prepared by blending in hydrophilic polymer polyvinyl alcohol (PVA) at 140 °C. A series of PVDF/PVA blend membranes was prepared at different total polymer concentrations and blend ratios. The morphological analysis via SEM indicated that the formation mechanism of these hydrophilically-modified membranes was a combined NIPS and TIPS process. As the total polymer concentration increased, the tensile strength of the membranes increased; meanwhile, the membrane pore size, porosity and water flux decreased. With the PVDF/PVA blend ratio increased from 10:0 to 8:2, the membrane pore size and water flux increased. The dynamic water contact angle of these membranes showed that the hydrophilic properties of PVDF/PVA blend membranes were prominently improved. The higher hydrophilicity of the membranes resulted in reduced membrane resistance and, hence, higher permeability. The total resistance *R_t_* of the modified PVDF membranes decreased significantly as the hydrophilicity increased. The irreversible fouling related to pore blocking and adsorption fouling onto the membrane surface was minimal, indicating good antifouling properties.

## 1. Introduction

The worldwide problems associated with the shortage of clean water have driven the rapid development of waste water treatment technologies. Membrane-based processes received much attention. In particular, microfiltration (MF) and ultrafiltration (UF) are the most popular methods for portable water purification due to the high efficiency, low costs, ease of implementation and low environmental impact [1,2]. MF (pore size range of 0.1 μm–10 μm) and UF (pore size range of 0.01 μm–0.1 μm) membranes are effectively used in wastewater pretreatment for filtering organic micropollutants. Amongst the commonly-used materials for MF/UF applications, poly(vinylidene fluoride) (PVDF) is one of the most widely-used membrane materials in water treatment due to its excellent chemical and thermal stability, as well as high mechanical strength [3,4].

Unfortunately, its lower surface energy and relatively high hydrophobicity lead to the increase of the transmembrane pressure of the PVDF membrane, irreversible fouling and rapid flux decline compared to hydrophilic membranes when treating waste water containing organic pollutants [4]. Subsequently, deteriorating membrane performance over time and frequent interruption of operation for membrane cleaning are the major drawbacks for MF/UF applications, leading to significant energy consumption and high cost. Attention has been paid to improving membrane performance to mitigate membrane fouling and reduce operation cost [5]. Usually, fouling is caused by the deposition of organic and inorganic compounds on the membrane surface and into its pores. These compounds commonly result in the formation of a cake and gel layer, adsorption and pore blockage [5,6,7,8,9,10]. It is known that a hydrophilic surface offers a better antifouling property for the membrane [11,12]. A pure water layer could easily form on a highly hydrophilic membrane surface for preventing the adsorption and deposition of hydrophobic pollutants and, hence, can reduce fouling [4]. Therefore, hydrophilic modification of MF/UF membranes becomes the main method to improve anti-fouling properties [4,11]. Among different methods, blending modification by hydrophilic polymers is the most reported approach due to its simplicity and effectiveness.

In the literature, nonsolvent-induced phase separation (NIPS) [13,14,15,16] and thermally-induced phase separation (TIPS) [17,18] are mostly commonly adopted to prepare PVDF membranes. In general, the NIPS method requires complicated control of the solvent exchange rate by simultaneously varying several process parameters, such as the dope composition, additives, coagulation medium, quenching bath temperature and evaporation time, to obtain PVDF membranes with the desired morphology and good performance [19,20]. In comparison, the TIPS process is relatively simple for obtaining a membrane with higher overall porosity, better mechanical strength and a narrower pore size distribution [4,21]. However, since it is difficult to form an asymmetric PVDF membrane structure with a skin layer using TIPS [4], NIPS is more commonly used to fabricate the PVDF membrane. Thus far, the PVDF/hydrophilic polymer blend membrane prepared by the TIPS method has not been reported in the literature. In TIPS, the polymer is dissolved in the diluent at high temperature, and phase separation is induced by cooling the dope solution [22]. Hence, the selection of diluent is crucial for membrane formation, as it serves as both a nonsolvent for the polymer at the quenching temperature (e.g., room temperature) and a strong solvent at high temperature (i.e., membrane fabrication temperature) to induce the TIPS mechanism [22]. Moreover, the dissolution temperature of PVDF and membrane preparation temperature are normally higher than the melting point of PVDF. For example, 240 °C was used as the membrane fabrication temperature by Ji et al. [23]. However, at such a high fabrication temperature, the hydrophilic polymers, such as polyvinyl alcohol (PVA) and polyvinylpyrrolidone (PVP), could be partly oxidized, cross-linked, thermally decomposed or depolymerized [24], which will strongly influence the stability and repeatability of the dope solution. In other words, the high temperature of the TIPS process is not suitable for the fabrication of hydrophilically-modified PVDF membranes. In our previous work [25], a novel water-soluble diluent ε-caprolactam (CPL) was first used to dissolve PVDF and obtained homogeneous PVDF casting solutions at a much lower fabrication temperature of 130 °C by a nonsolvent thermally-induced phase separation (NTIPS) method, which has combined the advantages of NIPS and TIPS. With the appropriately-selected diluent for the PVDF polymer, the low solution temperature can substantially eliminate the effects of high temperature on the stability of the hydrophilic polymer used for fabricating hydrophilically-modified PVDF membranes with the desired pore structure and performance.

In this study, to obtain the desired membrane characteristics under simpler fabrication conditions, a nonsolvent thermally-induced phase separation approach (NTIPS) [25] is first used to prepare hydrophilically-modified PVDF/PVA blend membranes. ε-caprolactam is used as the diluent of PVDF at a fabrication temperature of 140 °C. The PVDF/PVA blending ratio and total polymer concentration of the casting solution are optimized. The pore structure and filtration performance of the PVDF/PVA blend membranes are studied. Bovine serum albumin (BSA) is used as a modelled foulant to study the fouling propensity of the membranes. The membrane fouling resistance analysis is conducted to confirm the advantages of hydrophilically-modified PVDF membranes via the NTIPS method.

## 2. Materials and Methods

### 2.1. Materials

The PVDF (Model: 1015) polymer was supplied by Solvay Co. Bovine (Shanghai, China) serum albumin (BSA, *M_W_* = 67,000, Biochemical reagents) was purchased from Aladdin Industrial (Shanghai, China). Polyvinyl alcohol (PVA, Model: 1788) and ε-caprolactam (CPL, 99.5%), both supplied by Aladdin Industrial, were used as the hydrophilic modification polymer and diluent, respectively.

### 2.2. Preparation of Hydrophilically-Modified PVDF Membranes

The PVDF, PVA and CPL were mixed in a container proportionately to prepare a casting solution. The composition of the solutions for various PVDF/PVA blend membranes is shown in Table 1. The solution was heated up in an oil bath under the protection of nitrogen at 140 °C and stirred at a constant speed of 120 rpm to form a homogeneous dope solution. The solutions were degassed at the preparation temperatures and then were rapidly casted on the glass plate by an automated high-temperature casting machine described elsewhere [25], which was preheated to 140 °C. The nascent membrane was quickly and smoothly immersed into a water coagulant bath (25 °C). After the nascent membrane was completely solidified, the membrane was transferred into a flowing water bath to remove residual diluent and subsequently stored in DI water before use.

### 2.3. Membrane Characterization

The membrane surface and cross-sectional morphologies were observed using a scanning electron microscope (Model: TM3000, Hitachi, Tokyo, Japan). The membrane samples were fractured in liquid nitrogen. All samples were coated with a thin layer of gold in standard high vacuum conditions before scanning.

The mechanical properties of the membranes were measured via tensile strength using a tensiometer (Model: 5542, Instron Corp., Boston, MA, USA). Five pieces of membrane samples under each fabrication condition were tested to ensure reproducibility.

Dynamic water contact angles of the membranes were measured with an angle meter (Model: JC2000D2, Shanghai Zhongchen Company, Shanghai, China) to evaluate the membrane hydrophilicity. DI water was dropped on the sample surface at five different sites. Repetition of water contact angle measurements was done with three membrane samples under the same fabrication conditions.

The membrane porosity was tested according to the method described in the literature [26]. The membranes were weighed when wet and were later dried in an oven. The porosity (*P_r_*) was calculated with the following equation:

(1) $$P_{r}=\left(W_{w}-W_{d}\right)/d_{w}A_{m}L_{m}\times 100\%$$

where *W_w_* is the weight of the wet membrane (g), *W_d_* is the weight of the dry membrane (g), *d_w_* is the water density (g/cm^3^) and *A_m_* and *L_m_* are the membrane area (cm^2^) and thickness (cm), respectively.

The pore size distribution of the membranes was determined by the liquid-liquid displacement method based on an isobutanol-DI water system. The detailed experimental procedure can be found elsewhere [27,28,29,30].

### 2.4. Antifouling Performance and Membrane Fouling Resistance Analysis

For membrane performance evaluation, a flat-sheet membrane testing cell (MSC 300, Shanghai Mosu Science Company, Shanghai, China) was used to measure water flux under a pressure of 0.1 MPa. The effective membrane area was 35 cm^2^. The water flux, *J*, was calculated by the following equation:

(2) J=V/(At)

where *J* is the water flux (L·m^−2^·h^−1^), *V* is the volume of permeated water (L), *A* is the effective membrane area (m^2^) and *t* is the filtration time (h).

The membranes were pre-pressurized by filtering DI water for 0.5 h until the flux reached a plateau, and then, three steps of filtration were performed. Firstly, a 30-min period of recording the initial water flux (*J_i_*) was measured with DI water at 0.1 MPa. Secondly, the BSA filtration test was carried out for 1 h, and the membrane fouling step water flux (*J_f_*) was measured by filtering the BSA (1 g/L) solution at 0.1 MPa. Thirdly, at the end of the BSA fouling run, membrane “physical” cleaning was carried out, and then the post-cleaning water flux recovered (*J_r_*) was measured with DI water at 0.1 MPa. For membrane cleaning, the membrane surface was flushed with DI water under stirring (200 rpm) condition for 5 h. Normalized fluxes in Steps 2 and 3, i.e., *J_f_/J_i_* and *J_r_/J_i_*, were used to evaluate the antifouling performances of the currently-developed membranes [31,32,33,34].

According to Darcy–Poiseuille’s law [35], in the filtration process, the membrane total resistance *R_t_* (m^−1^) can be divided into the intrinsic membrane resistance *R_m_* (m^−1^) and the fouling resistance *R_f_* (m^−1^). *R_m_* is the initial hydraulic resistance, calculated from Darcy’s law Equation (3) [35] using the initial water flux (*J_i_*) measured:

(3) $$J_{i}=\Delta P/\mu R_{m}$$

where Δ*P* (Pa) is the transmembrane pressure and *μ* (Pa∙s) is the viscosity of the feed solution.

For further interpretations, fouling resistance *R_f_* can be divided into a reversible resistance *R_revf_* (m^−1^) and an irreversible resistance *R_irrf_* (m^−1^) [36]. *R_irrf_* can be calculated applying Equation (4) to the recovered water flux (*J_r_*), whereas *R_revf_* is calculated according to Equation (5):

(4) $$J_{r}=\Delta P/\mu (R_{m}+R_{irrf})$$

(5) $$J_{f}=\Delta P/\mu R_{t}=\Delta P/\mu (R_{m}+R_{f})=\Delta P/\mu \left(R_{m}+R_{revf}+R_{irrf}\right)$$

where *R_t_* is the total resistance (m^−1^). *R_revf_* is due to concentration polarization and the formation of a cake layer on the membrane surface, removable by physical cleaning; *R_irrf_* is due to pore blocking, and adsorption and can only be suppressed by chemical cleaning [37], which however was not investigated in this study.

## 3. Results and Discussion

### 3.1. Investigation on Total Polymer Concentration

Newly-prepared membranes *S*_1_–*S*_6_ (Table 1) with different total polymer concentrations at a PVDF/PVA blend ratio of 8:2 were used to investigate the influence on membrane structure and filtration performance.

#### 3.1.1. Effect of Total Polymer Concentration on Membrane Morphology

**Figure 1:**
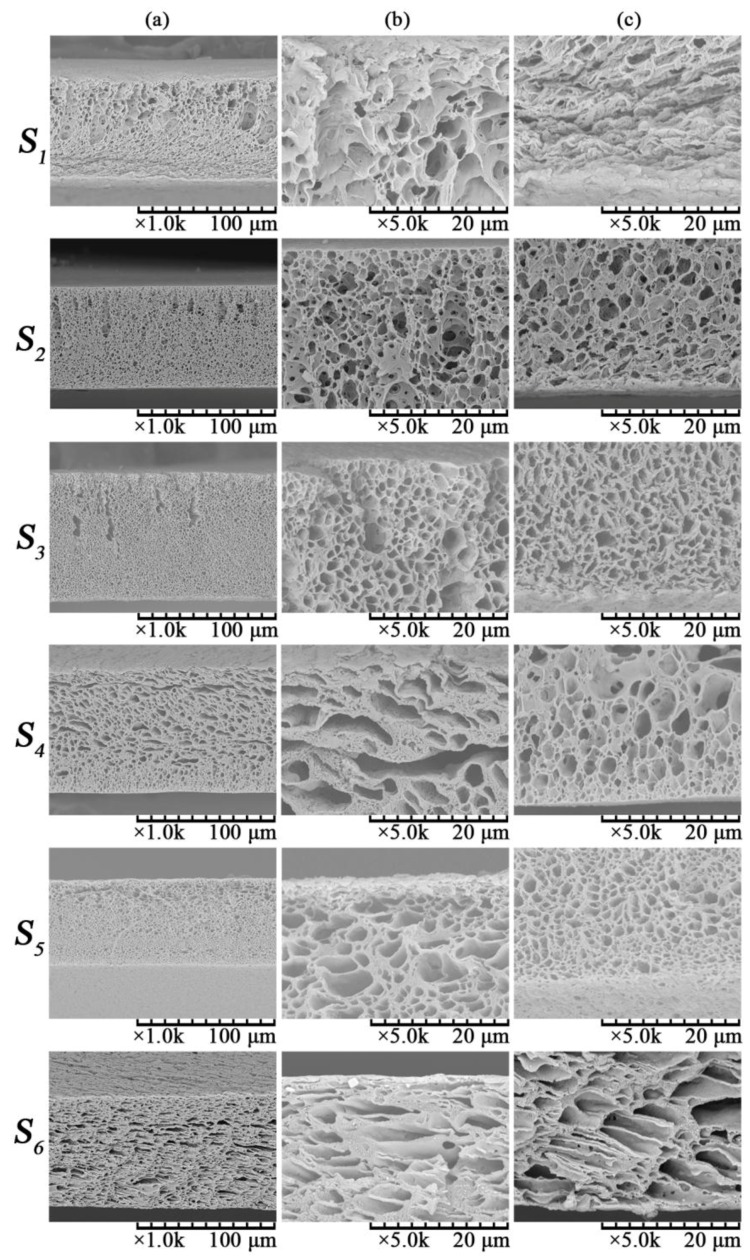
SEM images of PVDF/PVA membranes *S*_1_–*S*_6_: (**a**) full cross-section; (**b**) cross-section adjacent to the top surface; (**c**) cross-section adjacent to the bottom surface.

Figure 1 shows the cross-section morphologies of membranes *S*_1_–*S*_6_; Columns (a), (b) and (c) are the full cross-section, the cross-section adjacent to the top surface and the cross-section adjacent to the bottom surface, respectively.

It is observed in Figure 1b that the cross-section structure adjacent to the top surface gradually changes from long finger-like pores to cellular pores with increasing total polymer concentration from *S*_1_–*S*_6_. The finger-like pores eventually disappeared with the further increase in the total polymer concentration over 22 wt % (*S*_4_). This is because at a low total polymer content, the casting solution has low viscosity, and molecules move more freely [38], which is beneficial to the double-diffusion between water and solvent resulting in the formation of large finger-like pores. As the total polymer concentration increased to 22 wt % (*S*_4_) and above, both the viscosity of the casting solution and the mass transfer resistance of the solvent exchange increased. This has impeded the interactions between solvent and non-solvent (water) when the nascent membrane was immersed into the coagulation bath. The crystallization rate of PVDF would be reduced, hence resulting in the formation of a cellular pore structure adjacent to the top surface of the membrane. The NIPS mechanism plays an important role in forming the cellular morphology of the top surface, which was also observed in the literature [39].

Different from the cross-section structure adjacent to the top surface, Figure 1c shows that the cross-sectional structure in the sublayer adjacent to the bottom surface of the membrane gradually changes from a bicontinuous network pore to a cellular pore structure as the total polymer concentration increases from *S*_1_–*S*_6_. The pore structure of the bottom surface is mainly formed via the TIPS mechanism, as observed in a previous study [25]. Detailed explanations on the membrane formation mechanism will be provided in Section 3.2.

**Figure 2:**
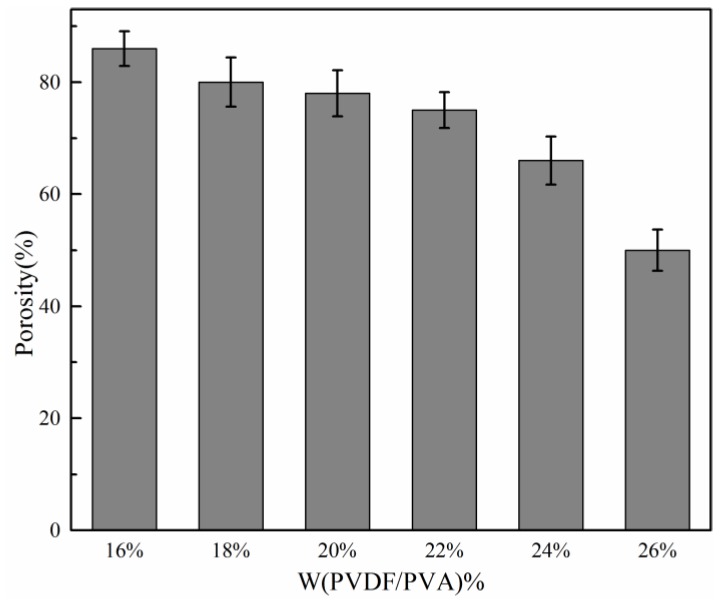
Effect of total polymer concentration on membrane porosity.

Figure 2 illustrates the effect of total polymer concentration on membrane porosity. It is observed that as the total polymer concentration increased from 16 wt %–26 wt % at the same blend ratio, the porosity of membrane decreased from about 86% down to 50%. The results are consistent with the variation in membrane structure indicated in Figure 1.

#### 3.1.2. Effect of Total Polymer Concentration on Membrane Pore Size and Pore Size Distribution

**Figure 3:**
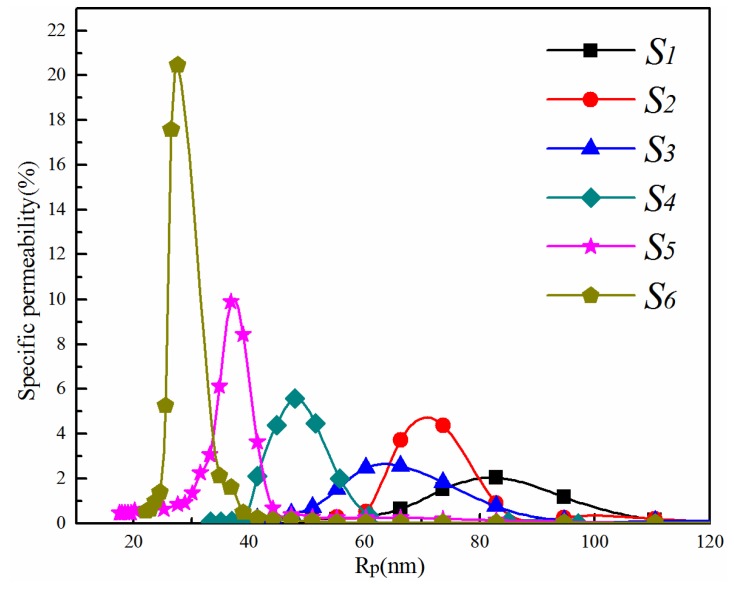
Pore size distribution curves of PVDF/PVA blend membranes *S*_1_–*S*_6_.

Pore size distribution curves and mean pore size of membranes *S*_1_–*S*_6_ are shown in Figure 3 and Table 2, respectively. With the increase of total polymer concentration, the curves of the membrane pore size distribution shift toward the left, and the mean pore size decreases. Overall, the width of the pore size distribution becomes narrower with increasing polymer concentration, indicating a more uniform pore structure. This corresponds to the decrease of the mean pore size from 81.7 nm down to 27.6 nm as the polymer concentration increased from 16 wt %–26 wt %. Membranes with UF (0.01 μm–0.1 μm) and MF (0.1 μm–10 μm) pore size ranges could be easily fabricated by adjusting the dope composition to meet the separation requirements.

#### 3.1.3. Effect of Total Polymer Concentration on Water Flux

**Figure 4:**
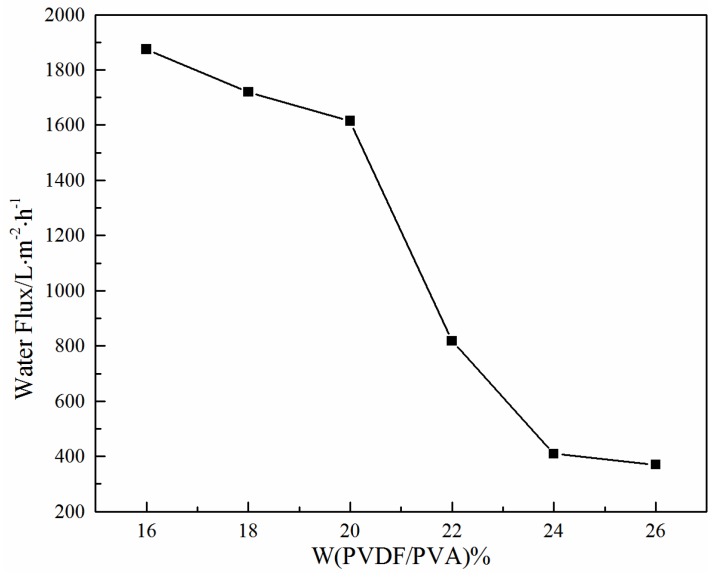
Effect of total polymer concentration on water flux for PVDF/PVA blend membranes *S*_1_–*S*_6_.

The effect of total polymer concentration on membrane flux is shown in Figure 4, in which the water flux of the membrane decreases with the increase of the total polymer concentration. This is mainly due to the decreasing pore size, as indicated in Table 2. At a total polymer concentration of 26 wt %, the water flux of the PVDF/PVA blend membrane *S*_6_ is about 370 L·m^−2^·h^−1^.

#### 3.1.4. Effect of Total Polymer Concentration on Membrane Mechanical Properties

**Figure 5:**
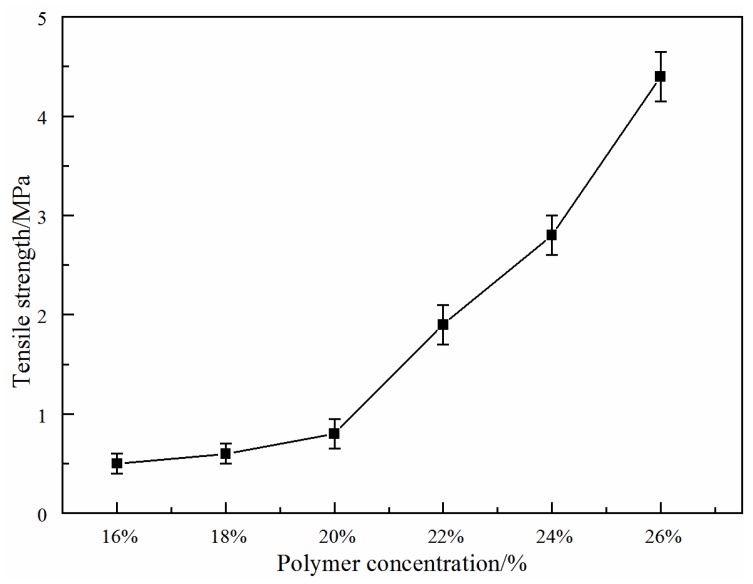
Effect of total polymer concentration on membrane tensile strength for PVDF/PVA blend membranes *S*_1_–*S*_6_.

Figure 5 shows the effect of total polymer concentration on the membrane tensile strength for PVDF/PVA blend membranes *S*_1_–*S*_6_. In general, the tensile strength of the membranes increases from 0.5 MPa–4.5 MPa with increasing total polymer concentration from 16 wt % (*S*_1_)–26 wt % (*S*_6_). The enhancement of mechanical strength is due to the change of the membrane structure from a long finger-like pore to a cellular pore structure, which led to decreased pore size and a narrower pore size distribution, as the total polymer concentration increased (Figure 1). This is also consistent with the decreasing porosity, as shown in Figure 2, which was also observed in previous work [25].

### 3.2. Investigation on the PVDF/PVA Blend Ratio

**Figure 6:**
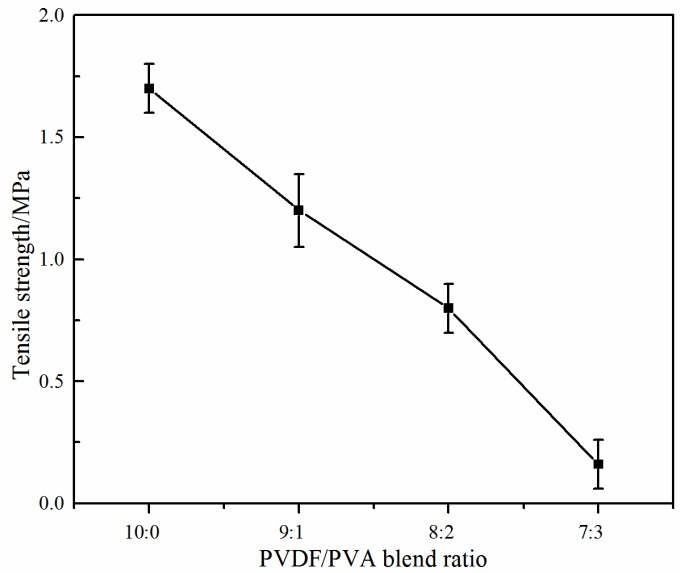
Effect of the PVDF/PVA blend ratio on tensile strength (total polymer concentration of 20 wt %).

With a pure PVDF membrane *N* as the benchmark, hydrophilically-modified PVDF/PVA blend membranes *M* and *S*_3_ (Table 1) with the same total polymer concentration of 20 wt % at varying PVDF/PVA blend ratios 9:1 (*M*) and 8:2 (*S*_3_) were fabricated for comparing the membrane structure and filtration performance. The effect of the PVDF/PVA blend ratio on the membrane mechanical properties is shown in Figure 6, in which the tensile strength of the blend membrane decreases from about 1.7 MPa down to 0.8 MPa with increasing blend ratio from 10:0 to 8:2. When the blend ratio of PVA further increased to 7:3 (membrane *L*; Table 1), the tensile strength of the PVDF/PVA blend membrane was as weak as 0.16 MPa, and the structure strength of blend membrane was poor for the performance test; hence, only the blend ratio lower than 7:3 was considered appropriate.

#### 3.2.1. Effect of PVDF/PVA Blend Ratio on Membrane Morphology

**Figure 7:**
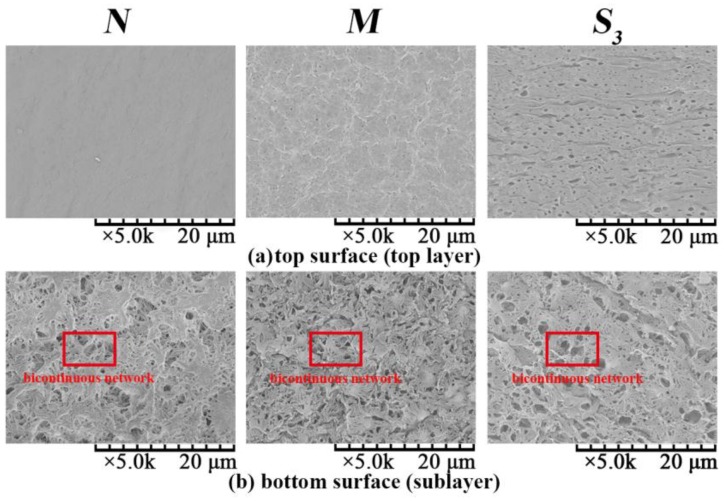
SEM images of membrane top and bottom surfaces of *N*, *M* and *S*_3_: (**a**) top surface (top layer); (**b**) bottom surface (sublayer).

The SEM images of the surface of membranes *N*, *M* and *S*_3_ are shown in Figure 7. The morphologies of the top surface (top layer) of PVDF/PVA blend membranes *M* (blend ratio 9:1) and *S*_3_ (blend ratio 8:2) are rough with microvoids; while the pure PVDF membrane *N* has a smooth top surface without observable microvoids. With increasing PVA content, the top surface becomes rougher and more porous. This is due to the enhanced integration between the interface of PVDF and PVA as the PVA content increases [11]. Meanwhile, all of the bottom surface (sublayer) morphologies exhibit a bicontinuous network porous structure and are significantly different from the top surface morphologies. The combined results from Figure 7a,b suggest that the top layer and the bottom layer of the membrane were formed by different phase separation mechanisms. Specifically, during the formation of PVDF/PVA blend membrane, TIPS and NIPS occurred simultaneously when the homogeneous dope solution was immersed into the coagulation bath of water, which serves as both a coagulant (in NIPS) and a coolant (in TIPS) . The solvent in contact with the outer surface of the casted layer (facing the coagulant side; Section 2) would exchange with water and then trigger the occurrence of the NIPS process, which determines the structure of the top layer (as shown in Figure 7a). Meanwhile, for the inner surface of the casted layer (facing the glass plate; Section 2), the heat exchange occurred between high temperature casting solution (140 °C) and coolant water (25 °C), inducing TIPS and subsequently the formation of the bicontinuous pore structure of the bottom sublayer (as shown in Figure 7b). The TIPS mechanism contributed heavily to the solid-liquid separation and crystallization in the polymer-rich phase and eventually to the formation of the network porous structure of the bottom surface of the membrane. This occurred because compared to the mass-transfer dominant NIPS process in the top layer, the heat transfer (dominant in TIPS) is much faster for forming the bottom surface [25]. This hence has explained the fundamental difference in the top and bottom surface morphologies.

**Figure 8:**
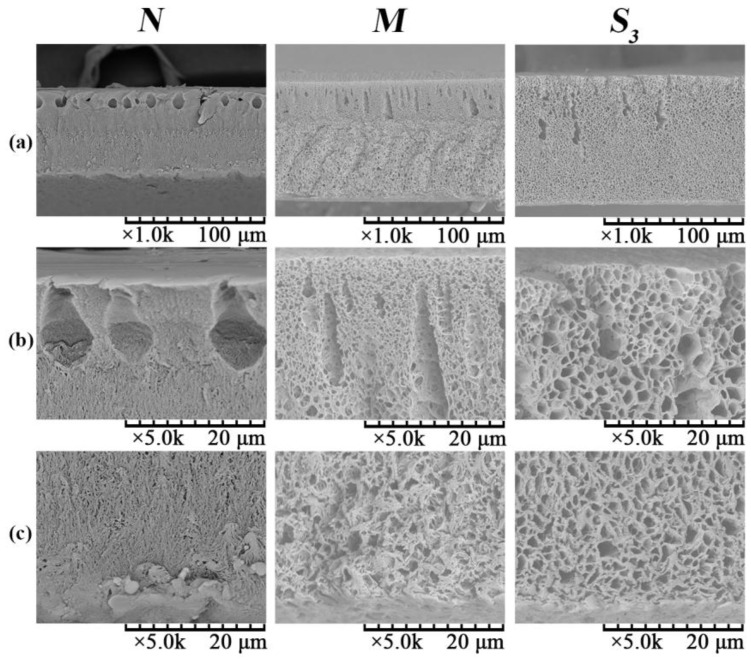
SEM images of membrane cross-section of *N*, *M*, *S*_3_: (**a**) full cross-section; (**b**) cross-section adjacent to the top surface; (**c**) cross-section adjacent to the bottom surface.

The SEM images of the cross-section of membranes *N*, *M* and *S*_3_ are shown in Figure 8; Rows (a), (b) and (c) are the full cross-section, the cross-section adjacent to the top surface and the cross-section adjacent to the bottom surface, respectively. Specifically, in Figure 8a, the finger-like pores beneath the top surface for the pure PVDF membrane *N* were formed mainly based on the NIPS mechanisms. This is because during membrane formation, the water (nonsolvent) quickly diffuses into the polymer solution beneath the top surface; hence, the rapid exchange rate of water and diluent CPL leads to the formation of the finger-like structure beneath the top surface. As the PVA content increases for the PVDF/PVA blend membranes *M* and *S*_3_, the number of the finger-like pores beneath the top surface decreases. This is due to the addition of hydrophilic PVA into the polymer dopes that increases the precipitation rate of the casting solution [14]. This may result in the formation of cellular morphologies via typical liquid-liquid (L-L) phase separation in the NIPS process [21]. Consistent with the observation in Figure 7, the cross-sectional morphologies adjacent to the bottom surface of all membranes exhibit a bicontinuous network porous structure. This is induced by the TIPS mechanism, in which the crystallization for macromolecules (i.e., PVDF) is much slower as a result of chain conformation compared to low molecular weight compound CPL. As CPL rapidly crystallizes at its original locations in a homogenous solution, this effectively suppresses the growth of PVDF crystals and subsequently dictates the pore structure. Thus, as CPL is dissolved in water, the space occupied by the polymer lean phase, or CPL-rich phase, becomes membrane pores with a well-connected bicontinuous network structure [25]. Hence, both surface and cross-sectional morphologies in Figure 1, Figure 7 and Figure 8 confirm that the formation mechanisms of the blend membranes are combined NIPS and TIPS, namely NTIPS.

#### 3.2.2. Effect of the PVDF/PVA Blend Ratio on Membrane Pore Size and Pore Size Distribution

**Figure 9:**
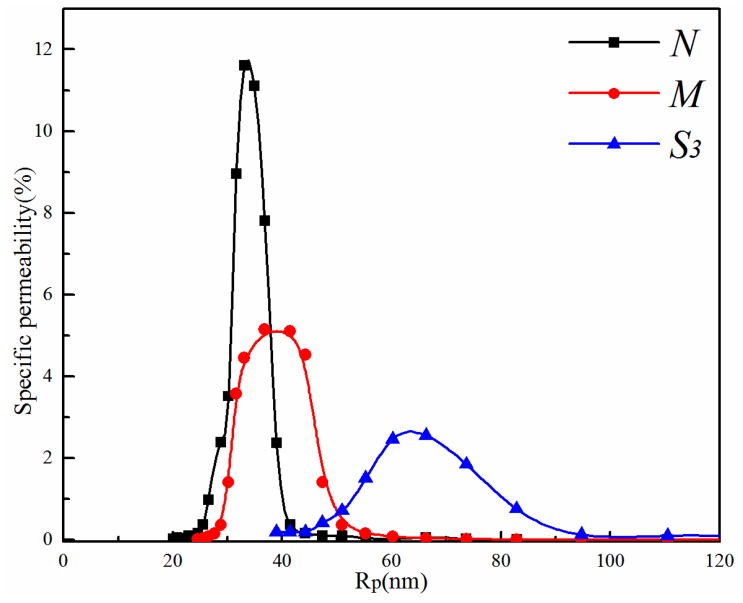
Pore size distribution curves of membranes *N*, *M* and *S*_3_ (total polymer concentration of 20 wt %).

The pore size distribution curves of membranes *N*, *M* and *S*_3_ are depicted in Figure 9, and the corresponding mean pore size is given in Table 3. As the PVA content increases, the pore size distribution becomes wider, corresponding to the increasing mean pore size from 34.3 nm (*N*)–64.6 nm (*S*_3_) in Table 3. This is consistent with the morphological observations from Figure 7 and Figure 8.

#### 3.2.3. Effect of the PVDF/PVA Blend Ratio on Water Flux

**Figure 10:**
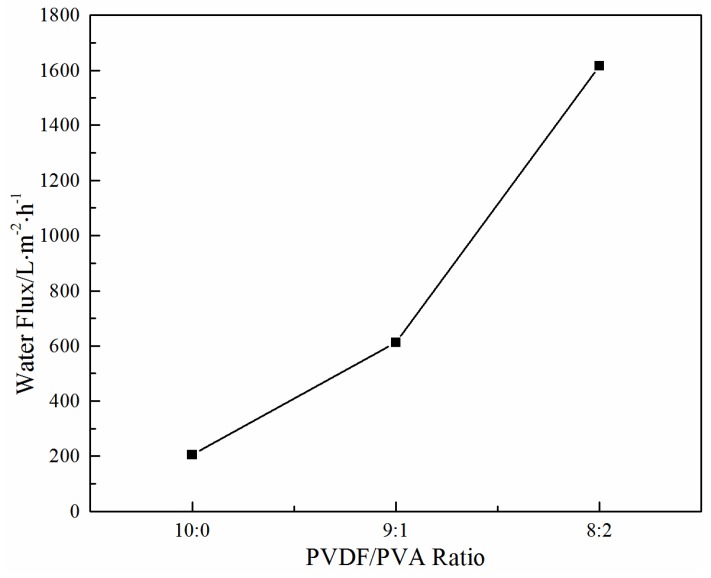
Effect of the PVDF/PVA blend ratio on water flux (total polymer concentration of 20 wt %).

Figure 10 illustrates the effect of the PVDF/PVA blend ratio on the water flux. As the PVDF/PVA blend ratio decreases from 10:0 to 8:2, the water flux increases significantly. The enhanced permeability is mainly due to the larger pore size (as shown in Table 3) and the wider pore size distribution with the increase of PVDF/PVA blend ratio. The results are also consistent with the structural variation of these membranes shown in Figure 7 and Figure 8, i.e., with the number of surface pores and pore size increasing, the water flux increases. The substantial improvement on membrane permeability is also attributed to the higher hydrophilicity of the blend membranes, which will be discussed in Section 3.2.4.

#### 3.2.4. Hydrophilicity of PVDF/PVA Blend Membranes

**Figure 11:**
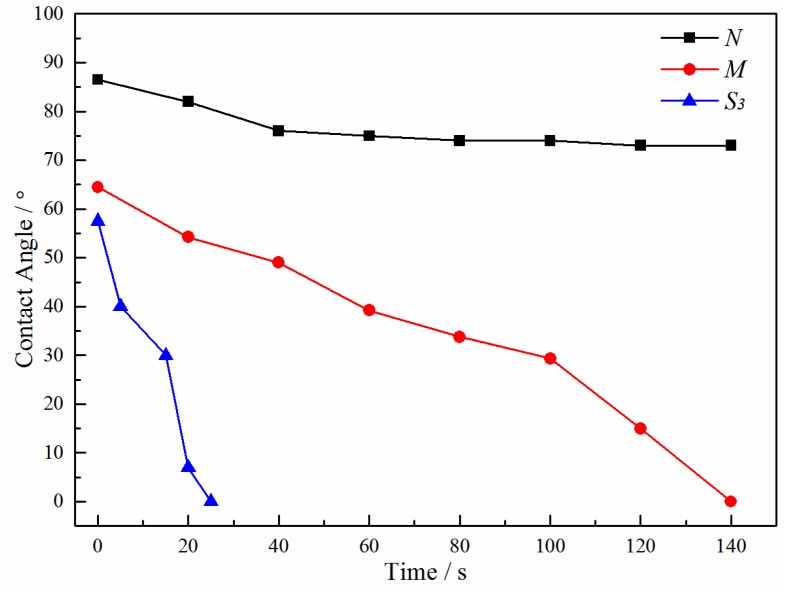
Dynamic water contact angles of membranes *N*, *M* and *S*_3_ (total polymer concentration of 20 wt %).

To study the membrane hydrophilicity, the dynamic water contact angles of the PVDF/PVA blend membranes *M* and *S*_3_ were measured and compared against the pure PVDF membrane *N*. The results are shown in Figure 11, in which the water contact angle decreases more significantly with increasing PVDF/PVA blend ratio from pure PVDF (*N*) to 8:2 (*S*_3_). For the control membrane *N*, the contact angle decreases from the initial value of about 86.5° and reaches a plateau of 73°; while that of the blend membrane *S*_3_ decreases most drastically from 57° down to 0° in 25 s, indicating complete penetration of water into the membrane matrix. Hence, it is obvious that the hydrophilic properties of PVDF/PVA blend membranes were prominently improved with the addition of PVA.

### 3.3. Antifouling Performance and Membrane Resistance Analysis

Hydrophilicity is an important factor affecting the fouling behaviour and filtration performance for MF/UF applications. The newly-prepared PVDF/PVA blend membranes *M* and *S*_3_ were selected to examine the antifouling performance, benchmarking against the pure PVDF membrane *N*.

**Figure 12:**
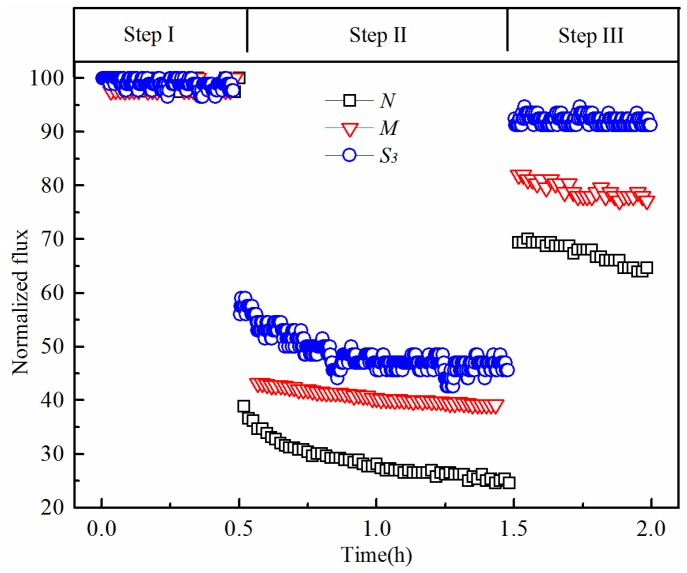
Antifouling performance of membranes *N*, *M* and *S*_3_ (total polymer concentration of 20 wt %).

As shown in Figure 12, the normalized fluxes *J_f_*/*J_i_* due to fouling and *J_r_*/*J_i_* after physical cleaning were used to evaluate the antifouling performance of the membranes. In the first filtration step as described in Section 2.4, the water flux (*J_i_*) declined slightly during the first 30 min for all tested membranes due to pre-pressurizing. However, in the second filtration step, the relative water flux (*J_f_*/*J_i_*) decreased significantly when the BSA solution was fed. The ratios of the water flux of the fouled membrane to their initial fluxes, i.e., *J_f_*/*J_i_*, of the membranes *N*, *M* and *S*_3_ reached plateau values of 24.7% (*N*), 39% (*M*) and 46.4% (*S*_3_), respectively. After cleaning with DI water in the third filtration, the water flux of all membranes was partially restored. However, the effect of physical cleaning on flux recovery is more efficient for the blend membranes *M* and *S*_3_. Specifically, for the pure PVDF membrane *N*, the water flux recovery ratio *J_r_*/*J_i_* stabilizes at 64.4% after cleaning; while for the hydrophilically-modified PVDF membranes *M* and *S*_3_, *J_r_*/*J_i_* are 77.6% and 91.1%, respectively. Hence, compared to the hydrophilically-modified membranes, the pure PVDF membrane is more susceptible to fouling caused by organic molecules, and the foulants stick to the membranes’ surface more tightly, which poses challenge for cleaning and long-term operation. The NTIPS PVDF membranes showed great potential for mitigating organic fouling by appropriately controlling the fabrication conditions.

**Figure 13:**
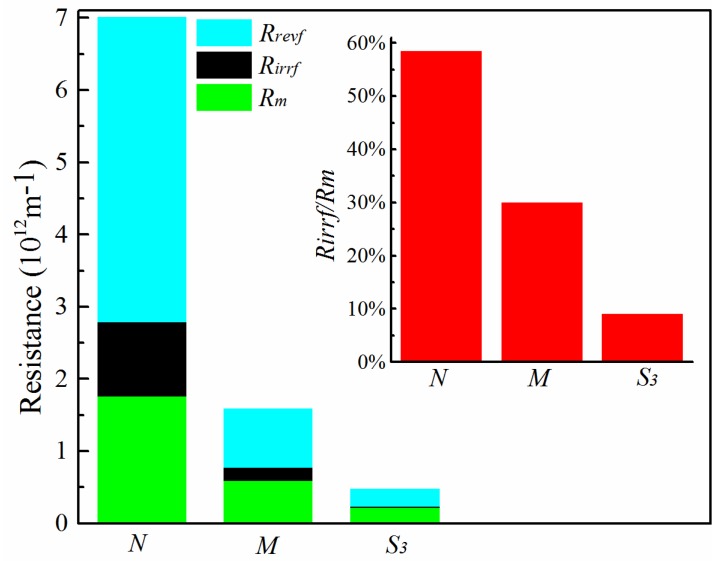
Fouling resistance analysis for membranes *N*, *M* and *S*_3_ (total polymer concentration of 20 wt %).

To further understand the fouling characteristics of the pure PVDF membrane *N* and hydrophilically-modified PVDF membranes *M* and *S*_3_, the local resistances *R_m_*, *R_revf_*, *R_irrf_* and *R_t_* are calculated based on Equations (3)–(5). The calculation results are shown in Table 4 and Figure 13. The total resistance *R_t_* of membranes *N*, *M* and *S*_3_ follows a decreasing order of 7.02 × 10^12^ m^−1^, 1.59 × 10^12^ m^−1^ and 0.49 × 10^12^ m^−1^, respectively. Thus, a significant reduction of *R_t_* was obtained for membrane *S*_3_ due to the higher PVA proportion. Consistent with the flux recovery results, this confirms the benefits of the hydrophilic modification for obtaining better filtration membranes with low fouling propensity and, hence, improved long-term performance.

Furthermore, as the PVDF/PVA blend ratio increases from 10:0 to 8:2, the value of *R_irrf_/R_m_* decreases from 58.5% down to 9.1%, as shown in Figure 13. Thus, the proportion of irreversible fouling resistance *R_irrf_* of the hydrophilically-modified PVDF membrane *S*_3_ is more significantly decreased compared to that of the control membrane *N*, resulting in less irreversible fouling, such as pore blocking and adsorption onto the membrane surface. Overall, the hydrophilic modification of the PVDF membranes exhibited good antifouling properties.

## 4. Conclusions

From this study, the following conclusions can be drawn:

(1) A novel nonsolvent thermally-induced phase separation (NTIPS) method was successfully employed to prepare hydrophilically-modified PVDF membranes. The PVDF/PVA blend membranes exhibited improved hydrophilicity, higher water permeability and enhanced fouling propensity.
(2) As the total polymer concentrations increased, the pore size, porosity and water flux of the PVDF/PVA blend membranes reduced, and the mechanical strength was improved. The membrane pore size could be deliberately tuned to meet separation requirements.
(3) Both surface and cross-sectional morphologies suggested that the formation of the hydrophilically-modified PVDF/PVA blend membranes was due to NTIPS mechanisms. Different from the top surface structure, which was mainly formed via the NIPS mechanism, the bottom surface of all membranes exhibited a bicontinuous network induced by TIPS.
(4) The dynamic water contact angle of the modified membrane dropped more rapidly indicating improved hydrophilicity with the addition of PVA. However, the ratio of PVA to PVDF should be carefully chosen with the considerations of membrane mechanical strength and filtration performance.
(5) Membrane resistance analysis revealed that the hydrophilically-modified PVDF membranes had lower total resistance of mass transfer (hence, higher permeability) and showed great potential for mitigating irreversible fouling. High performance MF/UF membranes with the desired pore size can be achieved by optimizing fabrication parameters in NTIPS. In future research, surface functionalization could be incorporated into this work to obtain advanced composite membranes with further improved hydrophilicity and antifouling properties for wastewater treatment.

## Figures and Tables

**Table 1:** Composition of casting solutions for various PVDF/PV blend membranes. CPL, ε-caprolactam.

Membrane ID	Total Polymer (PVDF/PVA) Concentration (wt %)	PVDF/PVA Blend Ratio	CPL Concentration (wt %)
*S*_1_	16	8:2	84
*S*_2_	18	8:2	82
*S*_3_	20	8:2	80
*S*_4_	22	8:2	78
*S*_5_	24	8:2	76
*S*_6_	26	8:2	74
*L*	20	7:3	80
*M*	20	9:1	80
*N*	20	10:0	80

**Table 2:** Mean pore size of PVDF/PVA blend membranes *S*_1_–*S*_6_.

Membrane ID (Polymer Concentration)	*S*_1_ 16 wt %	*S*_2_ 18 wt %	*S*_3_ 20 wt %	*S*_4_ 22 wt %	*S*_5_ 24 wt %	*S*_6_ 26 wt %
Mean pore size (nm)	81.7	73.6	64.6	48.0	36.2	27.6

**Table 3:** Mean pore size of membranes *N*, *M* and *S*_3_.

Membrane ID (PVDF:PVA Blend Ratio)	*N* (10:0)	*M* (9:1)	*S*_3_ (8:2)
Mean pore size (nm)	34.3	41.6	64.6

**Table 4:** Calculation of the membrane resistance for membranes *N*, *M* and *S*_3_.

Local Resistance	*N*	*M*	*S*_3_
*R_m_* (10^12^ m^−1^)	1.76	0.60	0.22
*R_irrf_* (10^12^ m^−1^)	1.03	0.18	0.02
*R_revf_* (10^12^ m^−1^)	4.22	0.81	0.24
*R_t_* (10^12^ m^−1^)	7.02	1.59	0.49
*R_irrf_*/*R_m_*	58.5%	30.0%	9.1%
